# Supplementary material for: Circulating Cell-Free mtDNA Contributes to AIM2 Inflammasome-Mediated Chronic Inflammation in Patients with Type 2 Diabetes
Source: Cells. 2019 Apr 8;8(4):328. doi: 10.3390/cells8040328 (PMC6524162; doi:10.3390/cells8040328)
Supplement: Supplementary file 1 [file cells-08-00328-s001.zip › Supplemental information.pdf]

Supplemental information

Circulating cell-free mtDNA contributes to AIM2 inflammasome-mediated chronic inflammation in patients with type 2 diabetes.

Jung Hwan Bae, Seung Il Jo, Seong Jin Kim, Jong Min Lee, Ji Hun Jeong, Jeong Suk Kang, Nam-Jun Cho, Sang Soo Kim, Eun Young Lee and Jong-Seok Moon

Supplemental Figure S1

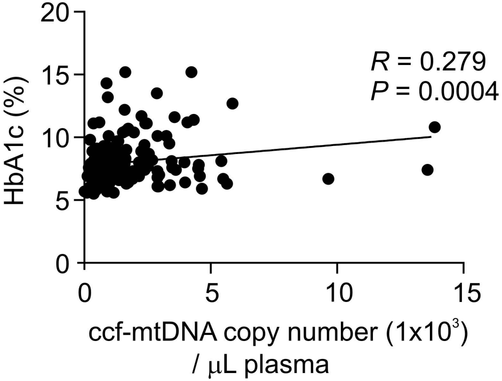

Supplemental Figure S1. The correlation between HbA1c levels and ccf-mtDNA levels in patients with type 2 diabetes. A Spearman correlation coefficient analysis to determine the relationship between HbA1c levels and ccf-mtDNA levels in plasma from 141 patients with type 2 diabetes. Data are mean ± SEM. *R*=0.279, *P*=0.0004 by Spearman correlation coefficient test. The black line shows the linear regression (r^2=0.0298).

Supplemental Figure S2

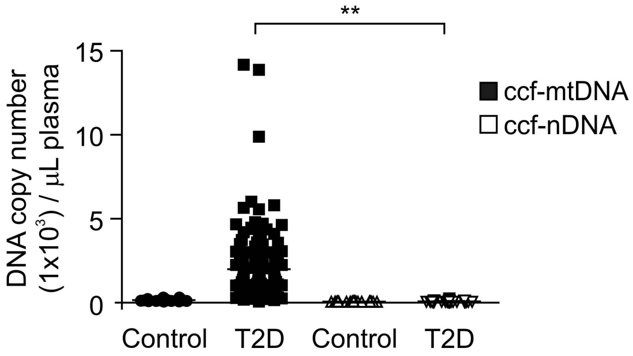

Supplemental Figure S2. mtDNA levels were higher than nDNA levels in ccf-DNA from patients with type 2 diabetes. Quantitative PCR analysis for mtDNA (ccf-mtDNA) and nDNA (ccf-nDNA) levels in ccf-DNA from plasma of 22 healthy subjects (control) and 141 patients with type 2 diabetes (T2D). Data are mean ± SEM. ***P*<0.01 by Student's two-tailed t-test.

Supplemental Figure S3

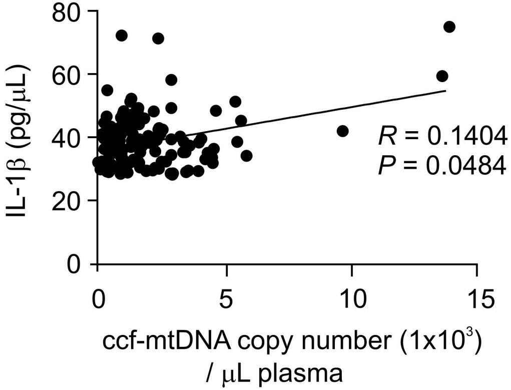

Supplemental Figure S3. The correlation between IL-1β levels and ccf-mtDNA levels in patients with type 2 diabetes. A Spearman correlation coefficient analysis to determine the relationship between IL-1β levels and ccf-mtDNA levels in plasma from 141 patients with type 2 diabetes. Data are mean ± SEM. R=0.1404, P=0.0484 by Spearman correlation coefficient test. The black line shows the linear regression (r^2=0.1107).

Supplemental Figure S4

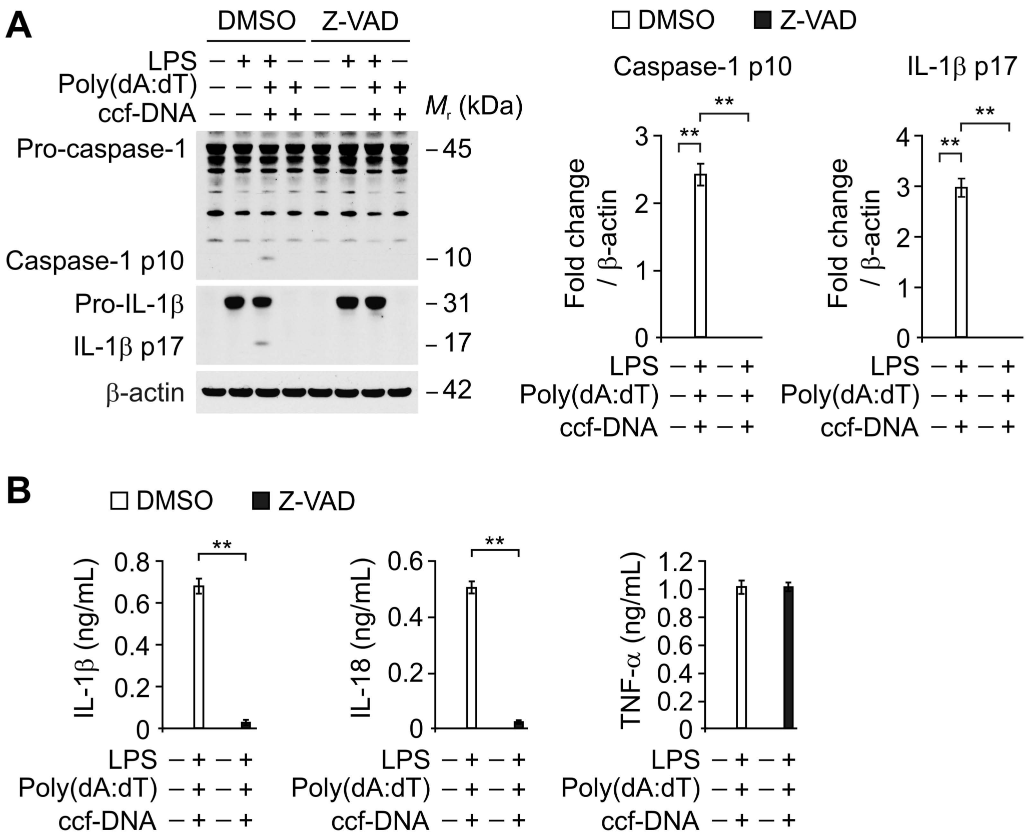

Supplemental Figure S 4. ccf-DNA-induced AIM2 inflammasome activation is required for caspase -1-dependent IL -1β and IL -18 secretion. (**A**) Representative immunoblot analysis for caspase-1 and IL-1β (left), and densitometry quantification of caspase -1 p10 and IL-1β p17 levels (normalized to levels of β-actin) (right) from WT BMDMs pre-treated with Z-VAD (10 μM, 1 h) before incubation with poly(dA:dT) and ccf-DNA after LPS stimulation For immunoblots, β-actin was used as loading control. (**B**) Quantification of IL-1β (left), IL-18 (middle) and TNF-α (right) secretion from WT BMDMs pre-treated with Z-VAD (10 μM, 1 h) before incubation with poly(dA:dT) and ccf-DNA after LPS stimulation. Data are mean ± s.d. ***P*<0.01 by two-tailed t-test. Data are representative of three independent experiments, and each was done in triplicate.
