# Supplementary figures and images for: Circulating Cell-Free mtDNA Contributes to AIM2 Inflammasome-Mediated Chronic Inflammation in Patients with Type 2 Diabetes
Source: Cells. 2019 Apr 8;8(4):328. doi: 10.3390/cells8040328 (PMC6524162; doi:10.3390/cells8040328)

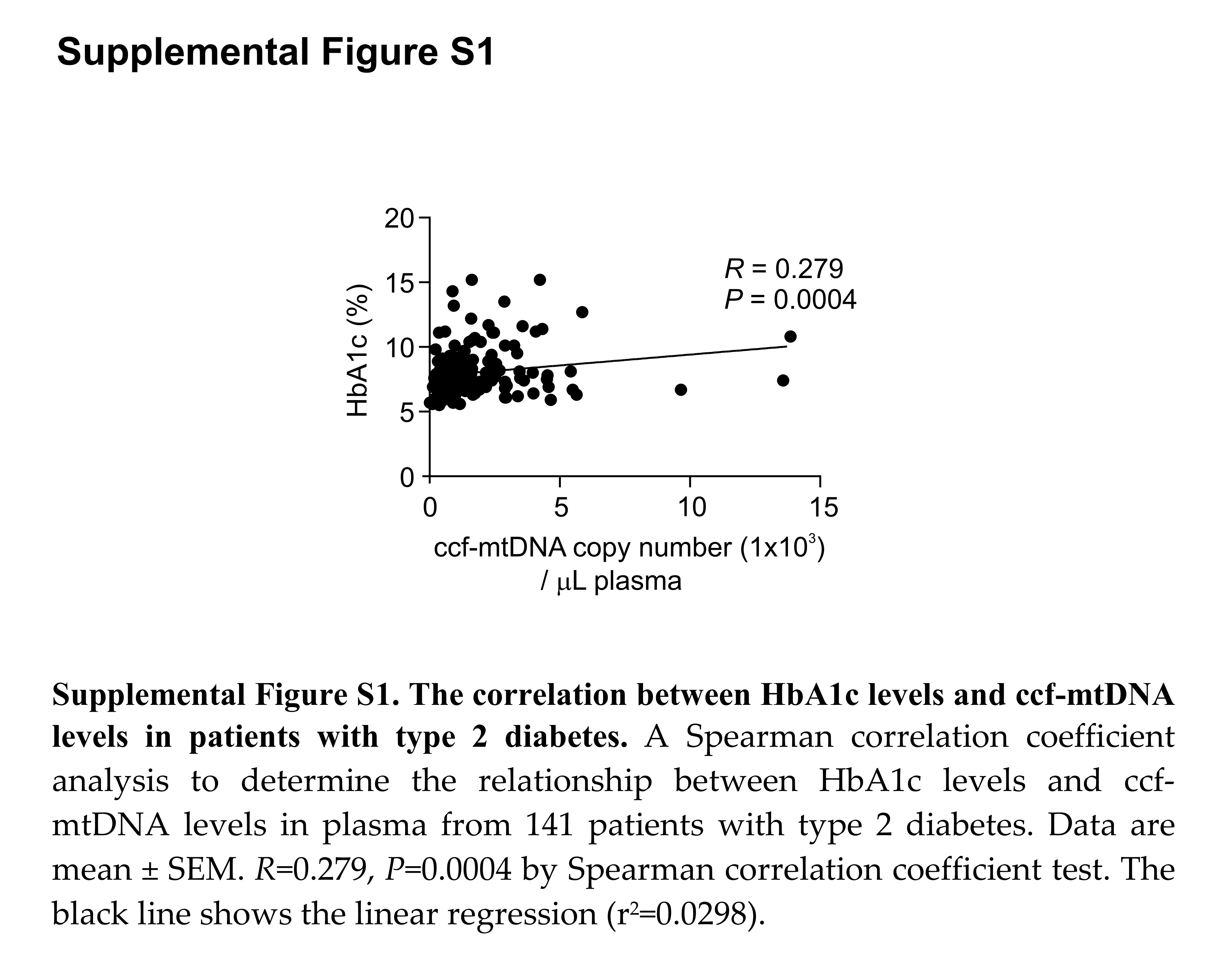

Supplement: Supplementary file 1 [file cells-08-00328-s001.zip › Figure S1.tif]

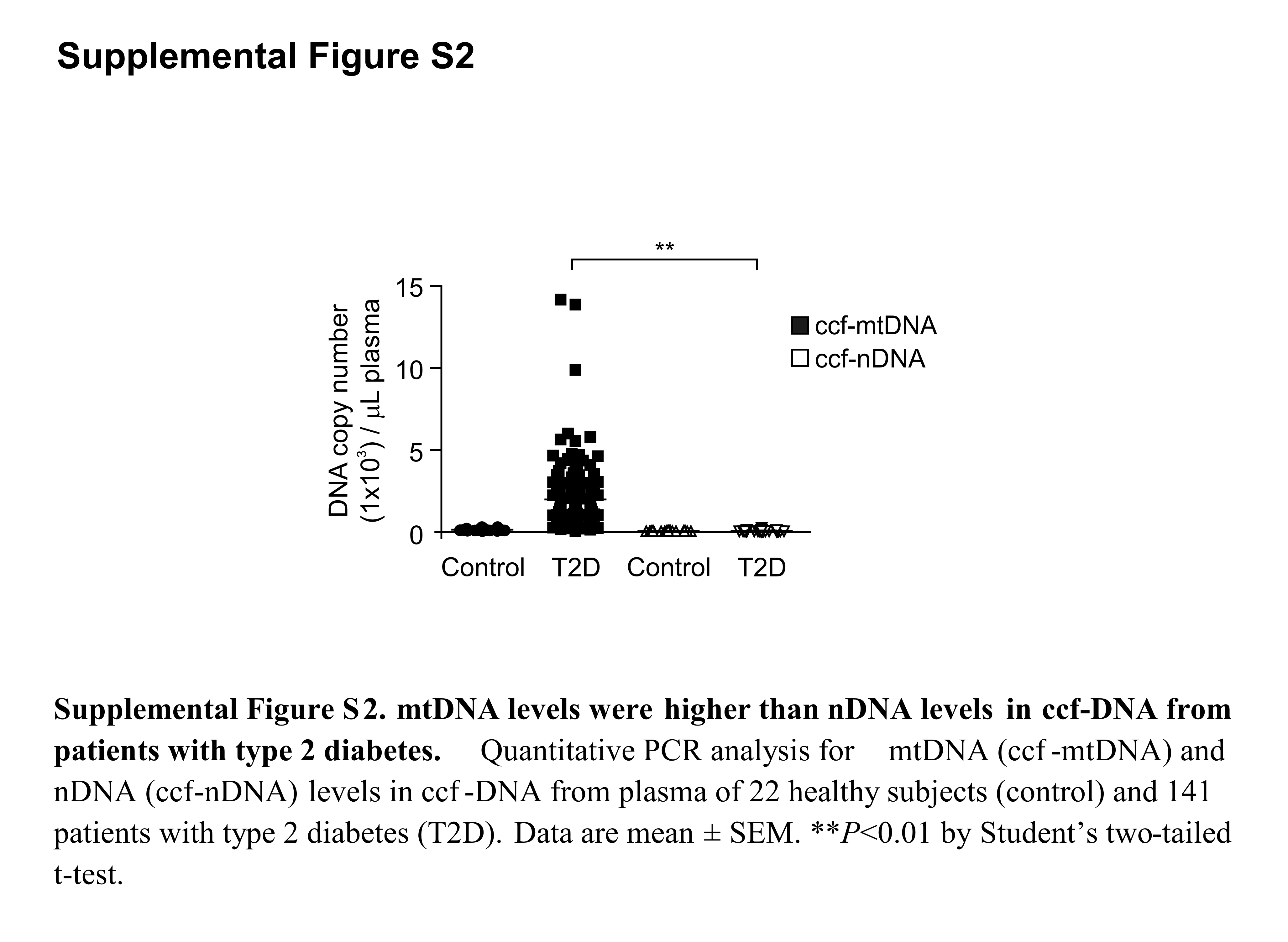

Supplement: Supplementary file 1 [file cells-08-00328-s001.zip › Figure S2.tif]

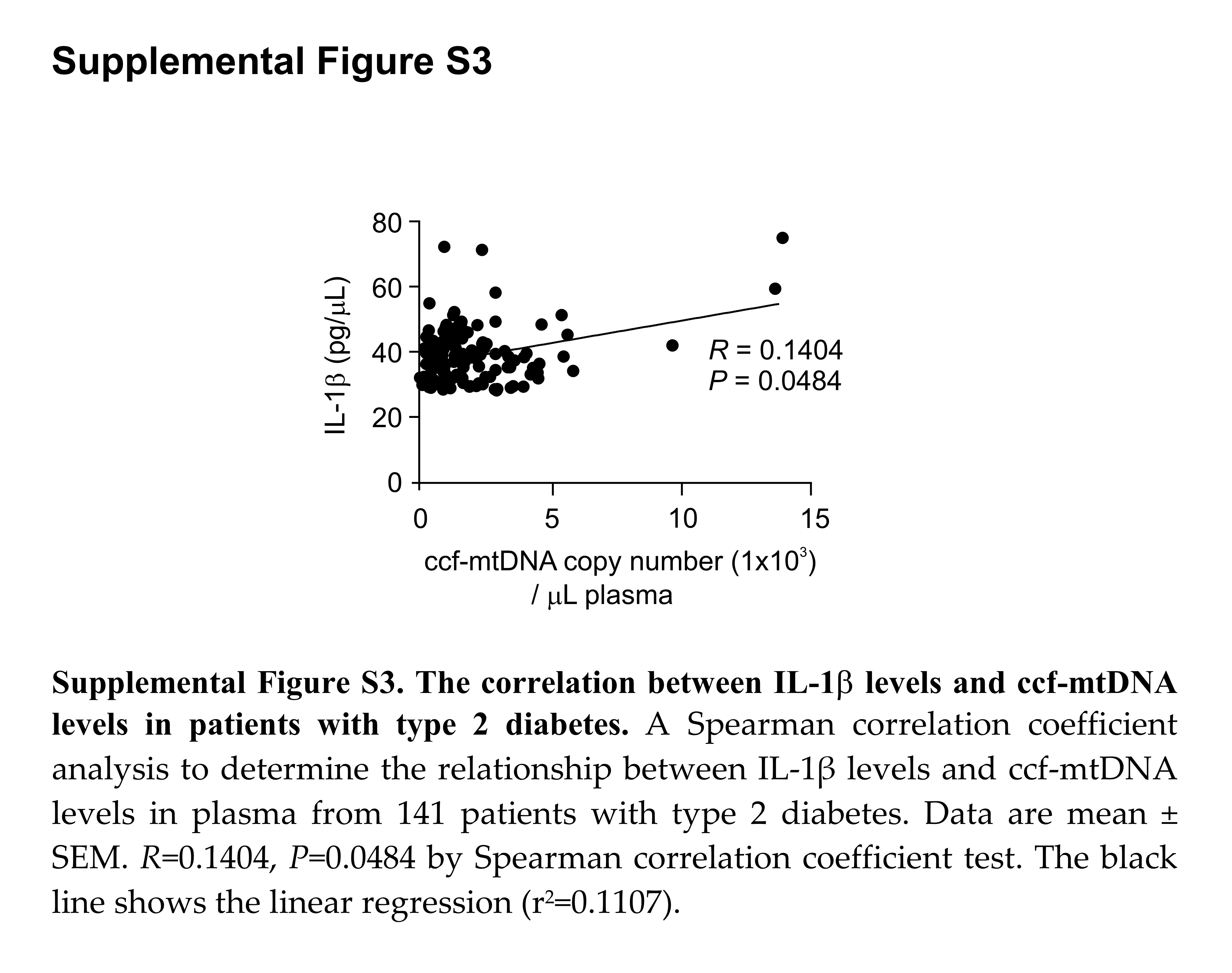

Supplement: Supplementary file 1 [file cells-08-00328-s001.zip › Figure S3.tif]

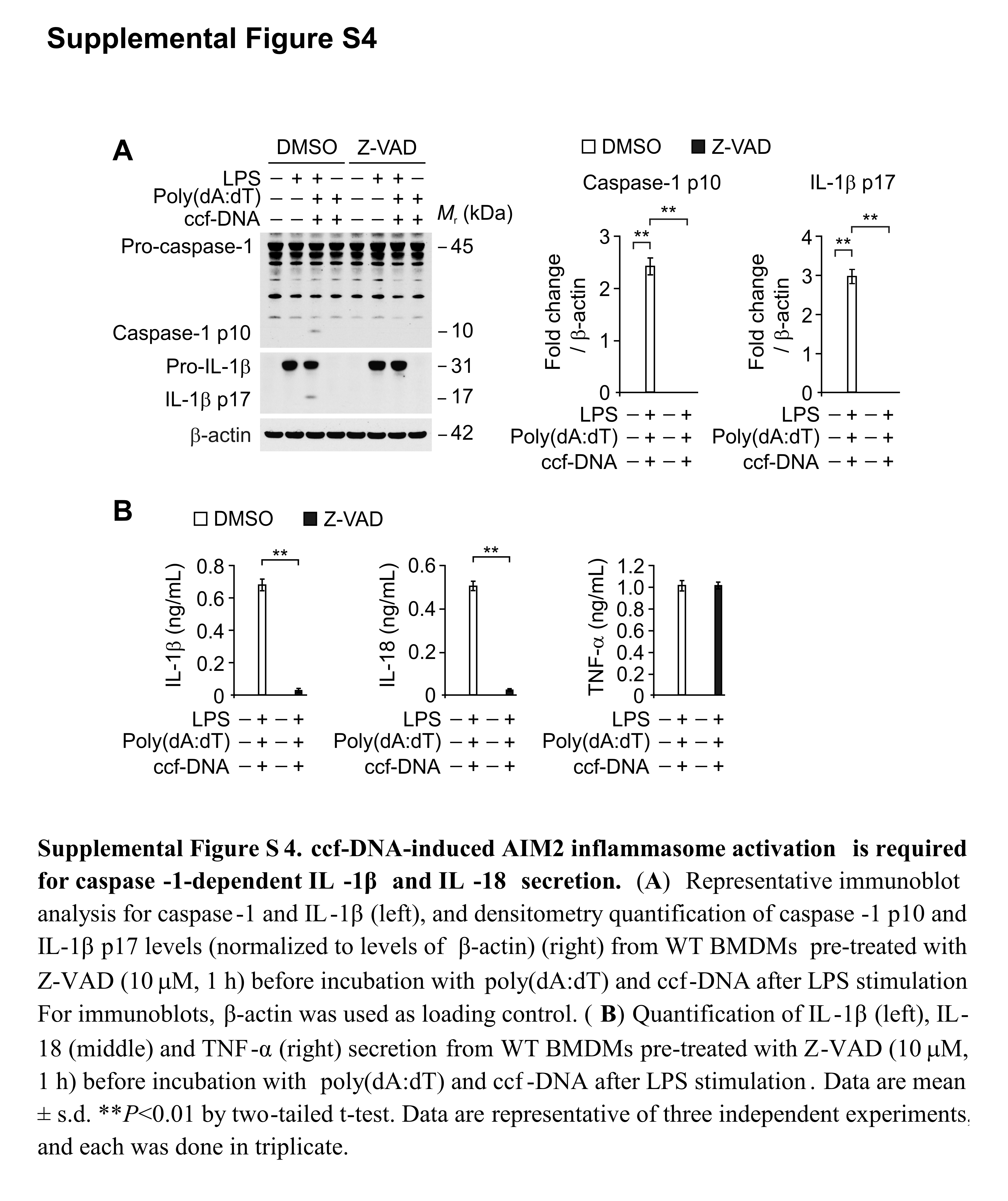

Supplement: Supplementary file 1 [file cells-08-00328-s001.zip › Figure S4.tif]
